# Knowledge about COVID-19 and Associated Factors Early in the Outbreak among the Brazilian Population

**DOI:** 10.3390/ijerph192113824

**Published:** 2022-10-24

**Authors:** Davi Amaral Cesario Rosa, Stéfanny Santos de Sousa, Murillo Nasser Rayol da Silva, Lauanda Raissa Reis Gamboge, Rodolfo Deusdará, Juliana Lapa

**Affiliations:** Faculdade de Medicina, Campus Universitário Darcy Ribeiro, Universidade de Brasília, Asa Norte, Brasilia 70873-100, DF, Brazil

**Keywords:** COVID-19, pandemics, knowledge, Brazil, cross-sectional studies

## Abstract

(1) Background: In Brazil, the first case of the novel coronavirus occurred on the 25 February 2020, and since then, it has spread rapidly over the entire country. During a pandemic, knowledge, attitudes, and practices are expected to largely influence the adherence to non-pharmacological interventions (NPIs). We evaluated the knowledge about COVID-19 and associated factors early in the outbreak among the Brazilian population. (2) Methods: A Brazilian cross-sectional study was carried out using an online questionnaire. The questionnaire consisted of the following topics: isolation, caring for someone sick at home, cleaning habits, disinfecting habits, and true and fake news. Logistic regression was conducted using sociodemographic and associated factors as the independent variables and a knowledge score as the dependent variable to estimate factors associated with knowledge about COVID-19. Crude, sex-, and age-adjusted odds ratios (OR) were calculated. (3) Results: Participants with a better educational status had higher odds of having a higher knowledge score (OR = 2.49, 95% CI = 1.15–5.37). Similarly, healthcare providers (health students and professionals) had higher odds of having higher scores regarding knowledge about COVID-19 (OR = 1.62, 95% CI = 1.05–2.48) than other counterparts. Of the wrong answers, the most frequent was the isolation period, followed by household recommendations to prevent COVID-19 and cleaning habits. (4) Conclusions: In conclusion, our study suggests that a higher educational status and being a healthcare provider are conditions associated with superior knowledge about COVID-19. In addition, inadequate knowledge related to isolation, COVID-19 prevention, and cleaning habits were found in our study. We believe that improving awareness to address these specific COVID-19 issues through a health education campaign is a significant approach for public health policymakers to fight against COVID-19 in Brazil.

## 1. Introduction

In December 2019 in Wuhan, China, a novel coronavirus was first detected and spread as an emerging respiratory pathogen [1]. On the 11 March 2020, the World Health Organization (WHO) officially declared the novel 2019 coronavirus disease (COVID-19) as a pandemic [2].

In Brazil, the first case occurred on the 25 February 2020, and since then, the epidemic has spread rapidly over the entire country. In response, the Brazilian Ministry of Health has adopted non-pharmaceutical interventions (NPIs), such as social distancing, lockdown procedures, universal masking, and handwashing [3]. However, the population’s commitment to government public policies, such as NPIs, was highly affected by knowledge, attitudes, and practices toward the disease. Previous Brazilian studies on dengue and Zika outbreaks showed a low level of adherence to preventive measures by individuals [4,5]. In addition, previous studies with the 2002 severe acute respiratory syndrome (SARS) and the 2012 Middle East respiratory syndrome (MERS) outbreaks showed that knowledge toward disease may influence population action [6,7]. 

During a pandemic, knowledge, attitudes, and practices are expected to largely influence the adherence to NPIs and may play an important role in avoiding and controlling the spread of the virus [8,9,10]. Knowledge surveys are used to diagnose knowledge gaps to implement public health education campaigns [11]. Often, the risk of infection and spread of the disease is related to the low level of knowledge of the disease, negative attitudes, and dangerous behaviors [12]. Beliefs and information about COVID-19 come to the population from governmental sources, social media, the internet, previous personal experiences, and medical sources [13]. The accuracy of those beliefs may determine different population behaviors about NPIs [13]. However, inaccurate information about COVID-19 is spreading faster than the virus, according to the WHO social media manager Aleksandra Kuzmanovic, and the dissemination of false information is a worldwide concern [14]. In Brazil, the main topic of fake news was related to prevention methods against COVID-19. WhatsApp is the primary channel for the dissemination of malicious content related to COVID-19 [15]. Consequently, the Brazilian Ministry of Health created a page on its official website aimed at clarifying fake news and providing the population with true information [15]. 

Based on the literature, healthcare workers and high socioeconomic individuals had a higher knowledge score for COVID-19 [10,13,16]. In addition, poor knowledge was related to misinformation spread through social media, and fake news issues were related to COVID-19 prevention and cleaning habits, as well as politics and COVID-19 statistics [15,17,18]. 

Considering that Brazil had not previously experienced the severe acute respiratory syndrome (SARS) epidemic in 2002 and the Middle East respiratory syndrome (MERS) epidemic in 2012, our aim was to evaluate knowledge about COVID-19 and associated factors early in the outbreak among the Brazilian population. We hypothesized that individuals of high socioeconomic status had a higher knowledge score for COVID-19, and a low knowledge score was related to misinformation of prevention methods against COVID-19. 

## 2. Materials and Methods

This was a Brazilian cross-sectional study carried out using an online questionnaire. Due to the COVID-19 lockdown restrictions, the sampling approach was based on convenience and a non-probabilistic snowball technique. This method was used because of the initial response and to recruit future subjects to participate in the study [19].

### 2.1. Pilot Study

We conducted an exploratory survey with 1 open question and 11 closed questions, covering 11 topics, such as virus information, transmission, symptoms, diagnosis test, preventive measures, mask and glove use, isolation, how to act if you are sick, attitudes when you are living with someone sick, and risk factors. Most of the respondents were interested in attitudes when you are living with someone sick (67%), treatments (47.8%), and virus information (40.3%). However, due to conflicting results about chloroquine use in that period, we avoided this issue.

Subsequently, we performed a pilot survey on 31 volunteers to test the acceptability of the COVID-19 knowledge questionnaire based on data from different protocols, including the Centers for Disease Control and Prevention (CDC), the World Health Organization (WHO), and the Brazilian Ministry of Health [20,21,22]. We received important feedback on two points: comprehension problems related to the use of medical vocabulary and complaints about the questionnaire length.

Based on these suggestions, the final questionnaire was developed with 16 questions covering topics such as daily life related to living with someone sick: isolation (1.a to 1.c), caring for someone sick at home (1.d to 1.e), cleaning habits (1.f to 1.h), disinfecting habits (2.a to 2.c), and true and fake news (2.d to 2.h).

### 2.2. Recruitment of Participants

Participants were recruited between 16 May and 26 May 2020. An online electronic questionnaire was created using the “Google Forms” platform and posted into the interviewers’ WhatsApp groups. The first page of the survey consisted of a brief introduction, including the objectives and procedures of the study; terms of participation, clarifying voluntary adhesion; and terms of privacy and confidentiality. All participants agreed to participate and signed a free and informed consent form. To avoid duplicate reporting, we used the “Google Forms” settings to limit one completed form per participant. In addition, each completed form had a unique email address. During the cleaning data step process, 7 forms were deleted, 6 forms were foreign respondents, and 1 was a duplicated email address. Of the 447 forms completed, 7 forms were excluded, and 440 forms were our final sample. 

### 2.3. Data Collection

The questionnaire consisted of two sections. Firstly, assessment of sociodemographic aspects, individual aspects, and contact with COVID-19, and secondly, knowledge about COVID-19 (16 questions). The first section consisted of questions addressing sociodemographic aspects (sex, age, state of residence, and level of education) and contact with COVID-19. The second section consisted of 16 true/false questions regarding knowledge about COVID-19. A correct answer scored 1 point and an incorrect one scored 0 points. The knowledge level ranged from 0 to 16. A higher score denoted better knowledge.

Once the questionnaires were filled in, the participants received links to videos developed by the authors based on the WHO, CDC and the Brazilian Ministry of Health protocols covering all items presented in the questionnaire [20,21,22].

### 2.4. Data Analysis

The normality assumption was evaluated using the Shapiro–Wilk test for age and knowledge score variables. As a result, the age variables (W = 0.93516 and *p*-value = 0.00), and knowledge score (W = 0.95386 and *p*-value = 0.00) were non-normally distributed.

For the descriptive analysis, absolute and relative frequencies were calculated for categorical variables, and measures of central tendency (mean) and variation (standard deviation) for continuous variables with normal distribution. For the other variables, medians and interquartile ranges were calculated.

The COVID-19 knowledge score system ranged from 0 to 16, and it was categorized in two groups, characterized by low and high scores. A high score was considered ≥14 out of 16.

Logistic regression was conducted using sociodemographic and associated factors as the independent variables and a knowledge score as the dependent variable to estimate factors associated with knowledge about COVID-19. Crude, sex-, and age-adjusted odds ratios (OR) were calculated along with 95% confidence intervals (CI).

All analysis was conducted in STATA version 14 (StataCorp LP, College Station, TX, USA). *p*-values of 0.05 were considered significant.

## 3. Results

A total of 440 Brazilian participants completed the questionnaire and almost 75% of whom were women. The median age was 34 years of age, with 50% of the sample between 24 and 45 years of age. More than half of the participants were living in the Central-West region, followed by the Southeast region with approximately 27%. More than half of the participants have completed higher education and postgraduate studies. Less than 2% did not complete twelve years of education.

Information from the questionnaire showed that (Table 1) more than a half of the participants were not healthcare providers (meaning health students and professionals). Furthermore, less than 7% of individuals reported a suspected or confirmed infection with COVID-19, but approximately 74% of the respondents knew someone diagnosed with COVID-19. The majority were not at high risk for COVID-19, but more than half of the participants lived with people who were at high risk.

The median score was 14 (IQR = 2) out of a total of 16 (Table 1).

The data related to the score in the questionnaire can be seen in Table 2. The percentage of correct answers ranged from 68% to 97%. 

Of the wrong answers, almost 30% of individuals believed that: (1) there is no minimum time of isolation for household members (Table 2, question 1.b), (2) they did not need to wear gloves when in contact with objects used by an infected person (Table 2, question 1.h), (3) the clothes of diseased people should be hand washed (Table 2, question 2.b), and (4) smokers were not at increased risk of developing severe forms of disease (Table 2, question 2.d). In addition, almost 20% believed that alkaline foods such as garlic, lemon, and vitamin C helped prevent COVID-19 (Table 2, question 2.f).

The odds ratio (adjusted and unadjusted) between socio-demographic factors, associated factors, and knowledge about COVID-19 can be seen in Table 3. After multiple adjustments, participants with a better educational status had higher odds of having a higher knowledge score (OR = 2.49, 95% CI = 1.15–5.37). Similarly, healthcare providers had higher odds of having higher knowledge scores (OR = 1.62, 95% CI = 1.05–2.48) than other counterparts.

## 4. Discussion

COVID-19 is spreading rapidly and increasing exponentially in Brazil [23]. Online surveys are a promising method for assessing knowledge during outbreaks [18,24], particularly in low-income countries, given that data can be collected during the confinement period and be employed as a rapid screening method [8]. Often, online surveys were submitted by participants via social media, mainly Facebook, WhatsApp, and/or Instagram [18].

In our study, we evaluated knowledge about COVID-19 among the Brazilian population. The median score was high, which implies that a significant proportion of the participants are knowledgeable about COVID-19. This result was in line with previous studies [16,25,26,27]. It is worth emphasizing this study was conducted two months after the first index case was diagnosed in Brazil.

To our knowledge, this is the first study to investigate knowledge about COVID-19 in the general Brazilian population. Other studies have been conducted with specific segments, such as the elderly, diabetics, or dental health professionals [28,29,30].

The present study showed that higher educational levels were associated with higher knowledge scores for COVID-19. Participants who held an academic degree have higher exposure or access to multiple sources of information with accurate scientific information from reliable sources [25,31]. Knowledge about the disease is important because susceptibility to misinformation is associated with vaccine hesitancy and reduced likelihood of complying with public health guidance [32].

In our study, being a health student or professional (healthcare providers) was associated with greater knowledge about COVID-19, reinforcing the results already found in the literature [10,33]. Healthcare providers have better access to scientific information, training programs, and health research information [34]. Knowledge is a prerequisite for promoting positive behaviors, attitudes and prevention measures [35]. Good awareness among healthcare workers of transmission routes and preventive beliefs is essential to control the disease [16]. In previous studies, knowledge directly affected attitudes among health workers to defeat the virus [10,33], as well as medical students [36].

The most frequent wrong answers in our study were related to the isolation period, followed by household recommendations to prevent COVID-19 and cleaning habits. During the initial phase of the COVID-19 outbreak, the main issues related to fake news on Brazilian social media were homemade methods to contain the spread of COVID-19, followed by homemade methods to cure the disease according to the platform Eu Fiscalizo [15]. According to two official websites, politics, COVID-19 statistics, and prevention were the most commons issues [17]. WhatsApp and Facebook were the most common sources of misinformation [15,17]. Poor knowledge due to misinformation can negatively influence behaviors and attitudes, and people may fail to recognize and avoid high-risk situations for COVID-19 infection [15,17,18].

We did not find an association between knowledge about COVID-19 and suspected or confirmed disease cases, people belonging to high-risk groups, people knowing someone who had been diagnosed with COVID-19, and people living with someone who belonged to high-risk groups. That result was different from the previous literature, which demonstrated that having relatives or acquaintances with COVID-19 enhances awareness about the disease itself and the prevention measures [37,38]. One possible explanation for our null results is that psychological conditions, not considered in this study, such as anxiety, fear, and worry, could play an important role in this relationship with knowledge about COVID-19 [38].

There are some limitations related to our study. First, this was a convenient sample of the Brazilian population. Although, there were residents from each of the five main geographic regions (North, Northeast, Central-West, South and Southeast), more than a half were residents from the Central-West region. Thus, these issues may limit the representativeness of our study. Second, we used a non-validated questionnaire, as there was no knowledge survey validated at the moment that the study was conducted. However, this questionnaire was constructed based on recommendations from different protocols, including the WHO, CDC and Brazilian Ministry of Health [20,21,22]. On the other hand, we conducted an exploratory survey and performed a pilot study, which can be seen as strengths in our study.

## 5. Conclusions

Our study suggests that higher educational status and being a healthcare provider (meaning health students and professionals) are conditions associated with higher knowledge about COVID-19 among Brazilians. In addition, inadequate knowledge related to isolation, COVID-19 prevention, and cleaning habits were found in our study. Due to the limitation in the sample’s representativeness, more studies are needed to investigate knowledge, attitude, and practices among Brazilians, particularly in lower socioeconomic and educational statuses. However, from the results, we believe that improving awareness to address these specific COVID-19 issues through a health education campaign is a significant approach for public health policymakers to fight against COVID-19 in Brazil.

## Figures and Tables

**Table 1:** Sociodemographic characteristics and factors associated with knowledge about COVID-19 among 440 Brazilian respondents.

Variables				
**Continuous**	**n**	median	interquartile range	
age	440	34	23.5 (1°Q)	45 (3°Q)
knowledge score	440	14	13 (1°Q)	15 (3°Q)
**Categoricals**	n	(%)	95% confidence intervals	
female	330	75	70.7	78.8
educational status				
<12 years of education	31	7	5.0	9.9
≥12 years of education	409	93	90.1	95.0
being a healthcare provider (meaning health students and professionals)	182	41.4	36.8	46.0
confirmed or suspected cases of COVID-19	28	6.4	1.7	2.3
knowing someone who has been diagnosed with COVID-19	328	74.5	70.2	78.4
belonging to a high-risk group for COVID-19	124	28.2	24.2	32.6
living with someone who belongs to a high-risk group for COVID-19	289	65.7	61.1	70.0

**Table 2:** Knowledge about COVID-19 among 440 Brazilian participants.

Questions/Options	True/False	n (Correct Answer, % of the Total Sample)
Question 1: Regarding the necessary care facing the COVID-19 pandemic, what attitudes are indicated? You can check more than one alternative.		
Isolation		
1.a People infected with SARS-CoV-2 and able to recover at home should avoid using shared spaces at the same time as other household members and eat meals in a separate room.	true	408 (92.73%)
1.b There is no minimum time of isolation for the residents of the house in case of a positive diagnosis among them. All household members should stay in isolation until the resolution of the symptoms.	false	302 (68.64%)
1.c If possible, confirmed and suspected cases should stay in a separate room. It is also recommended for them to use a separate bathroom.	true	431 (97.95%)
Caring for someone sick at home		
1.d If someone develops shortness of breath or chest pain, he or she should call a healthcare provider.	true	412 (93.64%)
1.e If someone develops a cough or fever, it is essential to call a healthcare provider. It is contraindicated to use paracetamol before consulting a doctor.	false	316 (71.82%)
Cleaning habits		
1.f Personal items, such as dishes and towels, can be shared between a sick person and household members.	false	426 (96.82%)
1.g The contaminated person’s clothes and sheets must be kept separately from the rest of the household.	true	371 (84.32%)
1.h It is necessary to wear gloves when in contact with objects used by the infected person.	true	306 (69.55%)
Question 2: What is correct to say about COVID-19? You can check more than one alternative.		
Disinfecting habits		
2.a For domestic cleaning, it is recommended to use a bleach solution (250 mL of bleach to 1.0 L of drinking water) to disinfect surfaces, such as house floors.	true	395 (89.77%)
2.b The clothes of people in isolation must be hand washed.	false	314 (71.36%)
2.c The use of water and soap or 70% alcohol is not enough to clean surfaces that are touched several times a day, such as counters, tables, and knobs.	false	399 (90.68%)
Fake news		
2.d Smokers are at increased risk of developing the severe form of the coronavirus.	true	340 (77.27%)
2.e The analysis of the virus structure does not reveal any similarities with other existing viruses, which is an indication that it may have been modified in laboratory.	false	398 (90.45%)
2.f Alkaline foods such as garlic, lemon, as well as vitamin C help prevent COVID-19.	false	357 (81.14%)
2.g Gargling with warm water, salt, and vinegar can fight the coronavirus in the first days of infection when it is restricted to the throat.	false	402 (91.36%)
2.h Imports from China should not be carried out at the moment as the virus may be present in the items.	false	393 (89.32%)

**Table 3:** Crude and adjusted ** odds ratios between sociodemographic, associated factors, and knowledge about COVID-19 regarding the 440 Brazilian respondents.

Variables	Logistic Regression	
	High vs. Low Score	
	Crude	Adjusted
educational status(≥12 years of education) ^ϕ,^^Π^	2.63 * (1.23–5.64)	2.49 * (1.15–5.37)
healthcare providers (meaning health students and professionals) ^λ,Ω^	1.81 * (1.23–2.69)	1.62 * (1.05–2.48)
confirmed or suspected case of COVID-19 ^λ^	0.72 (0.34–1.56)	0.81 (0.32–2.04)
belonging to a high-risk group for COVID-19 ^λ^	0.66 (0.39–1.12)	0.78 (0.43–1.41)
knowing someone who has been diagnosed with COVID-19 ^λ^	0.81 (0.48–1.38)	0.87 (0.5–1.49)
living with someone who belongs to a high-risk group for COVID-19 ^λ^	0.71 (0.47–1.06)	0.69 (0.46–1.04)

* *p* < 00.5; ** sex, age; ^Π^ adjusted by sex, age, and healthcare provider’s condition; ^Ω^ adjusted sex, age, and educational status; ^λ^ reference category no; ^ϕ^ reference category < 12 years of education.

## Data Availability

The data presented in this study are available upon request from the corresponding authors.
